# Do Dolls Resemble Their Makers?

**DOI:** 10.3389/fpsyg.2022.777346

**Published:** 2022-02-07

**Authors:** Miki Uetsuki, Misako Kimura

**Affiliations:** ^1^Department of Community Studies, Aoyama Gakuin University, Kanagawa, Japan; ^2^Department of Contemporary Liberal Arts, Aoyama Gakuin Women’s Junior College, Tokyo, Japan; ^3^Department of Childcare, Hakodate Junior College, Hokkaido, Japan

**Keywords:** doll-maker resemblance, object-maker resemblance, dog-owner resemblance, self seeks like, mere exposure effect, face recognition

## Abstract

Many often say that people resemble their pets or that the faces of manga characters and Buddha statues resemble those of their artists. Previous studies demonstrated that participants could match dogs with their owners, suggesting that pets resemble their owners. Other studies also demonstrated that people can match personal belongings, including inanimate objects, to their owners. However, it is unknown whether people tend to make objects that resemble themselves. In this study, we examined whether people tend to make objects that resemble themselves with dolls made of cloth as stimuli. The results demonstrated that people tend to project themselves into dolls, even in the case of amateur college students. The mere exposure effect or the algorithm “self seeks like” may be at play in not only people’s selection of pets but also their making of objects.

## Introduction

The view that “people tend to marry people who resemble themselves” has been supported by many previous studies (Griffiths and Kunz, 1973; Zajonc et al., 1987; Hinsz, 1989; Keller et al., 1996; Little et al., 2006; Wong et al., 2018; Tea-makorn and Kosinski, 2020). These studies consistently reported that couples have higher facial resemblance than non-couples.

People also say that people resemble their pets. Coren (1999) reported that women with long hair tend to prefer the lop-eared dogs, while those with the short hairstyles preferred the prick-eared dogs, consistent with non-academic reports that pets look like their owners. Roy and Christenfeld (2004, 2005) have shown that participants could match dogs with their owners, though this result was confined to purebred dogs. They also demonstrated that ownership period and resemblance did not show a significant correlation. Thus, it is presumed that the owners choose dogs that resemble themselves when they start rearing their pets. Payne and Jaffe (2005) have also examined the pet-owner resemblance for wide range of ages and races for both pet owners and participants, using only the faces of the dogs as signals for judges, and showed that participants matched the pets with their owners correctly more than would be expected from random guessing by Monte Carlo simulation. These studies suggest that pets resemble their owners.

Nakajima et al. (2009) used Japanese participants to test dog-owner resemblance. Because the dog owners of Roy and Christenfeld (2004, 2005) were in the US and those of Payne and Jaffe (2005) in Venezuela, the populations of dog owners studied were racially diverse. On the other hand, Japanese people are more homogeneous ethnically, allowing the elimination of ethnic factors. Nakajima et al. (2009) conducted two experiments to show that participants could match dogs with their owners correctly at levels above chance. The results of Nakajima et al. (2009) also suggest that the performance was independent of the gender of the judges, their reported confidence in their decision, their experience keeping dogs as pets, the number of years they kept dogs as pets, the breeds of dogs, and their affection for dogs. In addition, Nakajima (2013) has clarified which part of the face is critical for judgment of dog-owner resemblance. His results suggest that dogs and their owners resemble each other in the eye region.

Can people then match personal belongings, including inanimate objects, to their owners? Alpers and Gerdes (2006) and Stieger and Voracek (2014) examined this with cars. Alpers and Gerdes (2006) used photos of the sides of cars, and Stieger and Voracek (2014) used grayscale photos of the fronts, sides, and backs of cars as stimuli. Alpers and Gerdes (2006) showed that participants were able to match the cars to their owners at above-chance levels. On the other hand, Stieger and Voracek (2014) showed that participants were able to match the owners to the front views of their cars at above-chance levels, but not to the side or back views. In addition, Stieger and Voracek (2014) suggested that people could match dogs to their owners’ cars as well as to their owners. These studies suggest there are some cases in which people can match personal belongings, including inanimate objects, to their owners.

Why were the participants able to match cars to their owners? It may be because the front views of cars resemble human faces. Ichikawa et al. (2011) dealt with face-like objects, for example, electrical outlets, flowers, and houses. Some objects resemble human faces and the headlights of cars look like eyes (Windhager et al., 2012). Thus, matching cars to their owners might involve judging the resemblance between the face-like views of cars and the owners’ face.

In addition to couple, dog-owner, and car-owner resemblance, people sometimes note that the faces of manga characters resemble those of their artists or that the faces of Buddha statues resemble those of their makers, though this may be merely folk psychology or an offhand report just like those of dog-owner resemblances in the past. However, it is possible that people tend to make objects that resemble themselves if they have a tendency to choose such objects. People may reflect their faces in those of statues, portraits, or dolls when there is no specific model, even though they face technical limitations. In this study, we examined whether people tend to make objects that resemble themselves using cloth dolls as stimuli. In Experiment 1, we examined whether the participants could match dolls with their makers—them being able to match, however, does not directly imply doll-maker resemblance. In Experiment 2, we examined whether they could match the two based on facial resemblance.

## Experiment 1

Does the resemblance between people and objects exist not only in choosing something, but also when making something? For example, the makers may reflect themselves in the faces of drawings, statues, manga characters, and dolls in spite of technical difficulties under conditions of no model. This experiment examined whether people tend to make objects that resemble themselves using a procedure that was used by Nakajima et al. (2009). We used dolls that students made in the classes as stimuli. The students were all amateur doll makers and they made dolls for a puppet show.

### Methods

#### Participants

A total of 102 students (88 females and 14 males; average 18.66 ± 0.68 years old) from colleges in Hokkaido and Tokyo participated in the experiment voluntarily in classes. None of them were familiar with the students in the photos used in the experiment. The sample size was determined based on a power analysis (the calculated sample size was 88, when effect size (w) = 0.3, α error probability = 0.05, power (1 − β error probability) = 0.8, and *df* = 1). The photo judgment task was non-invasive, and written agreement was obtained from all participants.

#### Ethics Statement

This study was carried out in accordance with the recommendations of the Provisions of Experiments, Ethics Committee of Hakodate Junior College. The photos were permitted to be used in the experiment by the students that made the dolls. The protocol was in accordance with the Declaration of Helsinki and approved by the Ethics Committee of Hakodate Junior College.

#### Stimuli

**Figure 1:**
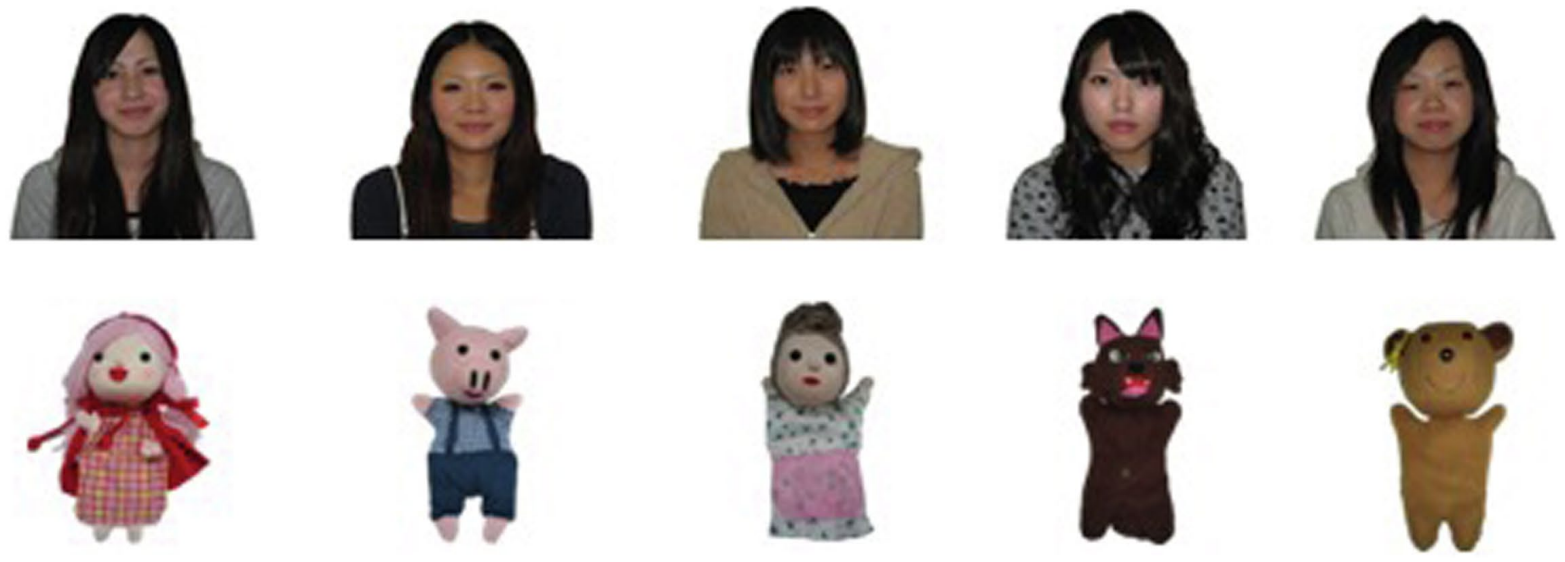
Examples of stimuli used in the study (dolls and their makers). For example, dolls of a little girl with a red hood, her grandmother, a hunter, and a wolf were made for “Little Red Riding Hood.” Printed with written permission from the makers.

Photos of 32 college students who had made a doll each for a puppet show in the class “Teaching Methods” for childminders and kindergarten teachers, and photos of those dolls were prepared as stimuli. They were all amateur doll makers, who made dolls for class credit. Of the 32 doll-maker pairs, 31 were female and one was male. Photos of the male student and his doll were excluded from the stimuli because they may have a gender effect (men may have less experience in sewing than women do. Thus, the dolls made by male students may differ from those made by female students in terms of perfection and fineness. It may be easy to judge whether the doll was made by a man or a woman. We excluded the photo of the male student and his doll to control such extraneous variables). When 31 pairs were divided into two sets, the number of pairs was different (15 pairs and 16 pairs). It is necessary to match the number of pairs because the difference may affect the judgments (for example, judgments about 16 pairs may be more accurate than those about 15 pairs). Thus, a female pair of photos with relatively low visibility was excluded from the stimuli. Thus, 30 doll-maker pairs were used in this experiment. The backgrounds of the photos were omitted using a graphics software (Adobe Photoshop). Figure 1 shows a sample of the stimuli used.

The photos of the 30 dolls and their makers were divided into two sets. One of the two sets comprised the “matching” pairs that had the correct (real) 15 doll-maker pairs. The other set contained the “mismatching” pairs that had the incorrect or mismatched 15 pairs, made by swapping the dolls’ photos within the 15 makers. The two sets were used for both the matching and mismatching pairs.

**Figure 2:**
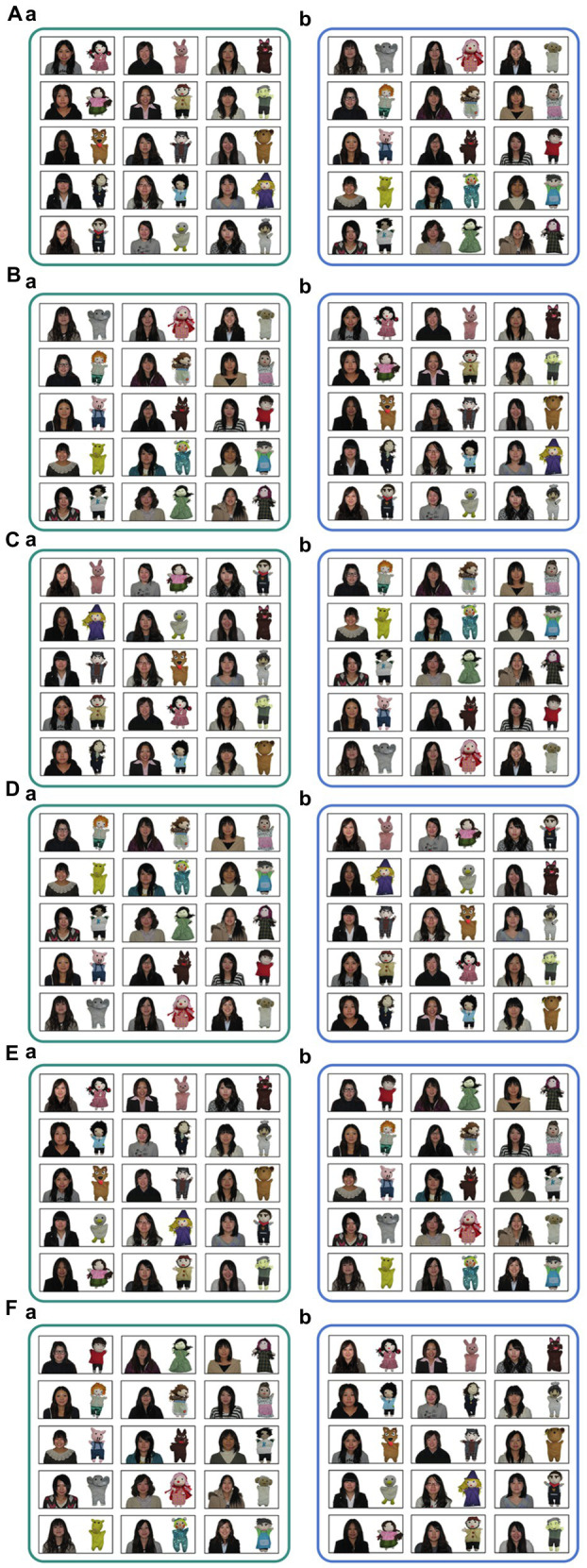
Examples of stimuli on the questionnaires. **(A)** A stimulus sample of 16 versions of questionnaires. Thirty doll-maker pairs were randomly divided into two, a and b. Each of the 15 pairs were surrounded by a colored rectangle. The left rectangle was green, and the right rectangle was blue. Each photo in a and b was randomly placed. Participants were required to judge which of the sets a or b had the matching (correct) doll-maker pairs. In this sample, the matching doll-maker pair was b. These 15 pairs were matched in half versions of the questionnaire. **(B)** A version with the left and right placements of **(A)** stimuli was swapped. In this sample, the matching doll-maker pairs set is a. **(C)** A sample of versions with the placement of **(A)** photos of each set were swapped line by line. For mismatching pairs, the combination of incorrect doll-maker pairs was also different from that in **(A)**. In this sample, the matching doll-maker pairs set was b. **(D)** A version with the left and right placements of **(C)** stimuli was swapped. In this sample, the matching doll-maker pairs set was a. **(E)** A version with the other 15 doll-maker pairs was matched, unlike in **(A–D)**. In this sample, the matching doll-maker pairs set was a. These 15 pairs were matched in half versions of the questionnaire. **(F)** A version with the left and right placements of **(E)** stimuli was swapped. In this sample, the matching doll-maker pairs set was b.

Each of the 15 pairs of photos was initially arranged in a random order in a 3 × 5 (row × column) matrix and each pair was surrounded by a black rectangle (Figure 2). The sizes of the photos of students were about 2.3 × 2.3 cm, those of the dolls about 1.2 × 2.3 cm. Both the matching and mismatching pairs were printed in color on a sheet of paper (297 × 420 mm); that is, a questionnaire consisting of a sheet of paper. The left–right placements of the matching and mismatching pairs were counterbalanced. The 15 pairs on the left were surrounded by a green rectangle and labeled “A,” and the other 15 pairs on the right were surrounded by a blue rectangle and labeled “B.” In order to prevent photo placements form affecting the results, multiple versions of the questionnaire were created by switching the placement of the doll-maker pairs line by line. Moreover, multiple combinations of the mismatched doll-maker pairs were randomly created. In this way, the photos’ positions were varied to yield 16 versions of the questionnaire (Figure 2 shows some examples of our questionnaires).

#### Procedures

One of the 16 versions of the questionnaire was distributed among the participants. The number of participants varied slightly between the 16 versions of the questionnaire because the questionnaires were randomly and blindly distributed to the participants. Each participant had to listen to and read the instructions. Discussion with other participants was strictly prohibited during the experiment. In addition, the experimenter confirmed that the photos printed on the questionnaire did not include any acquaintances of the participants.

If participants agreed to participate in the experiment, they were asked to fill their ages and genders on the questionnaire. After that, they had to make their final decision. Participants were only required to choose the matching doll-maker pairs, A or B. They answered by circling one of A or B on the questionnaire in a two-alternative forced-choice task (2AFC). The task took about 5 minutes including instructions.

### Results and Discussions

The participants’ correct answer rate for doll-maker pairs was 64.71% (the number of matching pairs choices was 66, and there were 36 incorrect choices); the difference was found to be significant using a chi-square test [*χ*^2^(1) = 8.824, *p* < 0.01]. Experiment 1 demonstrates that people can match dolls to their makers correctly at above-chance levels. The college students, who were amateur doll makers, made dolls as part of their class, and it was often the case that they could not make dolls as they had intended because of technical limitations. Nonetheless, participants were able to match the dolls with their makers correctly.

Previous studies used an ownership guessing task to show that pets resemble their owners. It is intuitively valid but semantically arguable because it is not certain that participants matched dogs to their real owners by resorting to dog-owner resemblance as a cue (Nakajima et al., 2009). Therefore, Nakajima et al. (2009) compared the performance on two task instructions, that is, a dog-owner matching task and a resemblance judgment task. The results suggest that the type of instructions had no effect on the participants’ choice. Even in the case of dolls, it may be expected that matching judgments in Experiment 1 were based on the resemblance between dolls and human faces. However, few studies, except for the one by Nakajima et al. (2009), have examined the effects of task instructions on facial resemblance. Therefore, in Experiment 2, we examined the effects of task instruction, that is, matching task and resemblance judgment task. Equivalent performance in both tasks is an indication of the participants thinking that the dolls look like their makers and are matching the former with the latter based on their resemblance. Differing performance in the tasks is an indication of the participants matching the dolls with their makers based on cues other than facial resemblance—in this case, high performance in the matching task (Experiment 1) does not mean that the dolls resemble their makers.

## Experiment 2

Experiment 1 shows that people can match dolls to their makers correctly. In Experiment 2, we examined whether the participants could match the dolls with their makers based on facial resemblance with a resemblance judgment task. Similar performance in both tasks being the same is an indication of the participants thinking that the dolls resemble their makers and matching the former with the latter based on their resemblance.

### Methods

#### Participants

A total of 128 students (84 females and 44 males; average age 19.7 ± 0.65) from a college in Hokkaido and of a university in Aichi participated in this experiment voluntarily in class. None of them were familiar with the students in the photos used in the experiment. The sample size was determined based on Experiment 1. The photo judgment task was non-invasive and written agreement was obtained from all participants.

#### Stimuli, Ethics Statement, and Procedures

The same stimuli as in Experiment 1 and 16 versions of the questionnaire were used. Participants made only one judgment, just as in Experiment 1. Experiment 2 required participants to choose the set of doll-maker pairs that resembled each other, A or B. Except for this instruction, the same procedure was used as in Experiment 1. Participants examined the resemblance between the doll-maker pairs in each set and answered by circling set A or B in which the pairs were more similar.

### Results and Discussions

The rate at which participants chose the matching doll-maker pairs as the resembling pairs was 64.84% (83 chose matching pairs and 45 chose the mismatched pairs); the difference was found to be significant using a chi-square test [*χ*^2^(1) = 11.28, *p* < 0.01]. Experiment 2 demonstrates that participants chose the correct doll-maker pairs as the resemblance pairs at above-chance levels. Table 1 summarizes the results of Experiments 1 and 2. Rates of choosing the matching pairs in Experiments 1 and 2 were nearly equivalent. This result demonstrates that people use doll-maker resemblances as a clue when matching dolls to their makers, as is the case with dogs.

**Table 1:** Results of Experiments 1 and 2.

	Matching task (Experiment 1)	Resemblance Judgment Task (Experiment 2)
Numbers of Participant	102	128
Numbers of choosing the matching pairs	66	83
Numbers of choosing the mismatching pairs	36	45
Rates of choosing the matching pairs	64.71%	64.84%
A Chi-Square Test (two-sided)	*χ*^2^(1) = 8.82, *p* < 0.01	*χ*^2^(1) = 11.28, *p* < 0.01

## General Discussions

In this study, we examined whether people make objects that resemble themselves. Experiment 1 showed that the participants could match the dolls with their makers, and Experiment 2 indicated that this matching was based on doll-maker resemblance. Even amateur makers (this study involved college students learning about childcare, who had made dolls for a puppet show for class credit) project themselves into their dolls regardless of whether it is humans or animal dolls. These results suggest that people prefer something that resembles themselves, not only when choosing objects but also when making them.

These results raise two questions. First, where is the resemblance evident. Second, why do the dolls resemble their makers? Nakajima (2013) demonstrated that dogs and their owners resemble each other in the eye region. However, the eyes of dolls are often simpler than those of dogs and humans, and it is not clear if they resemble them. This requires further study.

Why do they resemble each other? In the case of pets, while Roy and Christenfeld (2004, 2005) revealed that people select pets that resemble themselves, they do not provide the psychological mechanisms behind such selection. However, some accounts have been proposed by Hinsz (1989) and Payne and Jaffe (2005). In our study, we considered the mere exposure effect and the algorithm “self seeks like.”

Mere repeated exposure to a stimulus produces a more positive attitude toward it (Zajonc, 1968; Hinsz, 1989). Repeated exposure enhances attitudes toward a wide variety of stimuli, including the human face (Moreland and Zajonc, 1982; Hinsz, 1989). People are likely to be more attracted to faces they have seen most frequently (Moreland and Zajonc, 1982)—that is, their own face (through mirror exposure). Thus, mere repeated exposure would suggest that people prefer faces that resemble their own (Mita et al., 1977). It is through this process that they choose a pet or a partner whose face resembles their own. In addition, people make doll faces that resemble their own because those faces are more attractive.

“Self seeks like” is an evolutionarily shaped, narcissistic psychological algorithm and seems to be an innate behavioral trait in people—it characterizes assortative mating that seems to be widely practiced in nature (Alvarez and Jaffe, 2004; Payne and Jaffe, 2005). Assortative mating increases the probability of finding a genetically similar partner (Payne and Jaffe, 2005). If facial features are largely determined by genetic factors, we should detect assortative mating based on facial visual cues (Alvarez and Jaffe, 2004). Interestingly, it seems “self seeks like” is applied in situations where no reproductive purpose is involved (Alvarez and Jaffe, 2004)—Payne and Jaffe (2005) have suggested that people choose their pets by applying this algorithm.

It is not clear under what situations these algorithms would work, except when selecting objects. This study demonstrated that these algorithms work when creating objects. Unfortunately, we cannot conclude which mechanism is appropriate. However, it may be improbable that “self seeks like” works at creating objects because the algorithm is believed to be based on assortative mating, though it may be possible that the algorithm works at selecting objects.

It is often the case that we cannot make objects as we intend. For example, it is not easy for most people with big eyes to make dolls with big eyes because it requires skill or techniques, while it is easier for them to select dogs with big eyes. Nevertheless, our experiments demonstrated that even amateur doll makers, who made dolls for class credit, projected themselves into dolls unconsciously and made dolls that resembled themselves. This suggests that the mechanisms that people prefer objects that resemble themselves are powerful. This may be because the mechanism works repeatedly to make corrections and we have enough time to project ourselves when making objects over time.

This study demonstrated that people project themselves not only when selecting but also making something. If this is the case, statues of humans, including Buddha statues, and manga characters and their makers should show resemblances. This awaits further study, but the makers’ faces might be inferred from statues made some hundreds of years ago or from manga characters.

## Data Availability Statement

The original contributions presented in the study are included in the article/Supplementary Material, further inquiries can be directed to the corresponding author.

## Ethics Statement

The studies involving human participants were reviewed and approved by Ethics Committee of Hakodate Junior College. The patients/participants provided their written informed consent to participate in this study. Written informed consent was obtained from the individual(s) for the publication of any potentially identifiable images or data included in this article.

## Author Contributions

MU and MK conceived, designed, and performed the experiments at Hakodate Junior College and Aoyama Gakuin Women’s Junior College. MK analyzed the data. MU wrote up the study. All authors contributed to the article and approved the submitted version.

## Funding

This work was supported by JSPS KAKENHI Grant Number JP16K12516 to MK and MU. Open access publication fees were received from Aoyama Gakuin University.

## Conflict of Interest

The authors declare that the research was conducted in the absence of any commercial or financial relationships that could be construed as a potential conflict of interest.

## Publisher’s Note

All claims expressed in this article are solely those of the authors and do not necessarily represent those of their affiliated organizations, or those of the publisher, the editors and the reviewers. Any product that may be evaluated in this article, or claim that may be made by its manufacturer, is not guaranteed or endorsed by the publisher.
